# Analysis of m6A regulators related immune characteristics in ankylosing spondylitis by integrated bioinformatics and computational strategies

**DOI:** 10.1038/s41598-024-53184-z

**Published:** 2024-02-01

**Authors:** Da Guo, Jiayi Liu, Shuang Li, Peng Xu

**Affiliations:** 1 Osteonecrosis and Joint Reconstruction Ward, Department of Joint Surgery, HongHui Hospital, Xi’an Jiaotong University, Xi’an, 710054 Shaanxi China; 2 Xinglin College, Liaoning University of Traditional Chinese Medicine, Shenyang, 110167 Liaoning China; 3 Department of Neurology, Tangdu Hospital, The Fourth Military Medical University, Xi’an, 710038 Shaanxi China

**Keywords:** Computational biology and bioinformatics, Immunology, Diseases, Rheumatology

## Abstract

N6-methyladenosine (m6A) modification, as a common epigenetic modification, has been widely studied in autoimmune diseases. However, the role of m6A in the regulation of the immune microenvironment of ankylosing spondylitis (AS) remains unclear. Therefore, we aimed to investigate the effect of m6A modification on the immune microenvironment of AS. We first evaluated RNA modification patterns mediated by 26 m6A regulators in 52 AS samples and 20 healthy samples. Thereafter, an m6A related classifier composed of seven genes was constructed and could effectively distinguish healthy and AS samples. Then, the correlation between m6A regulators and immune characteristics were investigated, including infiltrating immunocytes, immune reactions activity, and human leukocyte antigen (HLA) genes expression. The results indicated that m6A regulators was closely correlated with immune characteristics. For example, EIF3A was significantly related to infiltrating immunocytes; IGF2BP2 and EIF3A were significant regulators in immune reaction of TGF-β family member, and the expression of HLA-DPA1 and HLA-E were affected by EIF3A and ALKBH5. Next, two distinct m6A expression patterns were identified through unsupervised clustering analysis, and diverse immune characteristics were found between them. A total of 5889 m6A phenotype-related genes were obtained between the two expression patterns, and their biological functions were revealed. Finally, we validated the expression status of m6A modification regulators using two additional datasets. Our findings illustrate that m6A modifications play a critical role in the diversity and complexity of the AS immune microenvironment.

## Introduction

Ankylosing spondylitis (AS) is a chronic autoimmune inflammatory disease characterized by radiographic sacroiliitis and positive axial spondyloarthritis. AS leads to general inflammation and significant pain, limited mobility and spinal deformity. The progression of AS may cause serious damage to spine, sacroiliac joint and peripheral joints^1,2^. AS is a complex multifactorial disease involving environmental factors, genetic susceptibility, and immunity. Studies have shown that the dysregulation or overactivation of the immune system plays a role in the pathogenesis of AS, with various immune cells, secretory mediators, and markers attracting wide attention^3^. For example, studies have demonstrated serum levels of IL-17 and IL-23, as well as an increased percentage of Th17 cells in AS patients^4,5^. IL-23 is essential for the expansion and maintenance of Th17 cells^6^. Yang et al. manifested the percentage of CD4+/CCR4+T cells were increased in active AS. CCR4, as a chemokine receptor of Th2 cells, is overexpressed on CD4+T cells to enhance Th2 response, while the immune regulatory function of Th2 cells may enhance Th1 response^7^. The immune regulation of AS is believed to be a key factor in its pathological mechanism, but the exact mechanism remains unclear and required further exploration.

In recent years, epigenetics has become a focus of research, playing an important role in elucidating the interaction between genes and the environment and altering the course of disease. Among various RNA modifications, N6-methyladenosine (m6A) modification is the most prevalent in epigenetic mechanisms and is present in eukaryotic specie ranging from yeast, plants, flies to mammals^8^. m6A modification is a dynamic regulatory process mainly determined by different m6A regulators, including methyltransferase (writer), demethylase (eraser) and binding protein (reader)^9^. Accumulating studies have indicated that m6A modification is involved in immune cell development, differentiation, activation and other processes, playing a role in immune response regulation. For example, m6A reprograms naive T cells for proliferation and differentiation by inducing degradation of Socs mRNAs in response to IL-7 signaling, thus regulating T cell homeostasis^10^. Other research suggests that after infection with the virus, DDX46 recruited ALKBH5 and eliminated the m6A modification, inhibiting interferon production and suppressing the innate response of antiviral^11^.Meanwhile, m6A modification has been reported to regulate immune cells to participate in the immune process of disease. For instance, in non-small cell lung cancer, METTL3 mediated m6A modification of circIGF2BP3 to inhibit CD8+T cell response through a decrease in PD-L1 ubiquitination promoting tumor immune escape^12^. These studies suggest that m6A RNA modification is closely related to the immune process of diseases, but its role in the immune mechanism of AS is rarely reported.

To address this gap, we systematically investigated the role of m6A modification and its related immune characteristics in AS. We found that the m6A regulators classifier could effectively distinguish healthy and AS samples. Immune characteristics such as infiltrating immunocytes, immune response activity and human leukocyte antigen (HLA) gene expression were significantly correlated with m6A modification. Next, we used the m6A regulators to cluster AS samples and identified two different m6A-mediated patterns. Additionally, these patterns were associated with different immune characteristics. We further studied the biological functions and key modules of m6A related expression patterns were studied through function enrichment and WGCNA analysis. The results suggest that the m6A modification pattern plays an important role in the immune properties of AS.

## Materials and methods

### Data collection

Three gene expression datasets of AS used in this study were downloaded from Gene Expression Omnibus (GEO) database (https://www.ncbi.nlm.nih.gov/geo/) using the “GEOquery” R package. Detailed information on the selected datasets is shown in Supplementary Table S1. GSE73754^13^ was applied to identify differentially expressed m6A regulators and analyze the related immune characteristics in AS. This dataset included 52 AS samples and 20 healthy samples, while GSE25101^14^ (16 AS samples and 6 healthy samples) and GSE181364^15^ (5 AS samples and 3 healthy samples) were used to validate the results. Based on the platform annotation file, the gene probes were annotated as gene symbols. Probes with multiple matching gene symbols or no matching gene symbols were excluded. The expression value of duplicate gene symbols was represented by the median value.

A total of 26 m6A modification regulators, including 9 writers (METTL3, METTL14, WTAP, KIAA1429, RBM15, RBM15B, CBLL1, ZC3H13 and ZNF217), 2 erasers (FTO and ALKBH5) and 15 readers (YTHDF1, YTHDF2, YTHDF3, YTHDC1, YTHDC2, HNRNPC, HNRNPA2B1, IGF2BP1, IGF2BP2, IGF2BP3, FMR1, ELAVL1, LRPPRC, EIF3A and EIF3B)^16–18^, were manually curated for this study based on previous publications.

### Alteration analysis of m6A regulators between AS and healthy samples

Differently expressed genes (DEGs) between AS samples and healthy samples were determined using “Empirical Bayes method” in the R package “limma”. A cutoff value of *P*-value < 0.05 was considered statistically significant. The intersection of m6A modification regulators and DEGs was calculated, and the overlapping genes were defined as the differentially expressed m6A regulators (DEMRs), which were considered AS-related m6A regulators. Volcano plot and heatmap were applied to visualize the DEGs and DEMRs between AS and healthy samples, respectively. Pearson correlation coefficient (PCC) was performed to assess the expression relationship of differently expressed m6A regulator genes (DEMRGs) in AS and healthy samples. The AS-related m6A regulators with a cutoff criteria of *P* < 0.05 were identified by univariate logistic regression. Subsequently, the least absolute shrinkage and selection operator (LASSO) regression was performed for feature selection and dimension reduction. An m6A regulator classifier of AS was developed using multivariate logistical regression. Finally, receiver operating characteristic (ROC) curve analysis was performed to assess the distinguishing performance of the classifier. Another dataset (GSE25101) was downloaded from the GEO database to validate the performance of the classifier.

### Correlation analysis between AS-related m6A regulators and immune characteristics

In this study, the relationships between AS-related m6A regulators and immune characteristics, including immunocyte subpopulations, immune reaction pathways, and HLA genes were analyzed. Gene sets related to immune cell subpopulations were obtained from a previous study^19^. Gene sets related to immune reaction pathways were downloaded from the ImmPort database (http://www.immport.org)^20^. Single-sample gene-set enrichment analysis (ssGSEA) using the “gsva” package of R was used to evaluate the scores of 28 types of immunocyte subpopulations and the activity of immune reaction pathways in all samples^21^. The expression levels of HLA genes were acquired from GSE73754 dataset. The enrichment scores of infiltrating immunocytes and immune reaction activity, as well as the expression level of HLA genes, were compared between AS samples and healthy samples using t-test. Pearson correlation analysis was used to calculate the correlations of m6A regulators with immunocyte fractions, immune reaction pathways and HLA gene expression. A correlation coefficient |r|> 0.3 was considered as significant, with a *P*-value < 0.05 indicating statistical significance.

### Analysis of m6A regulators related modification patterns

In order to identify different modification patterns of m6A regulators, unsupervised cluster analysis was conducted based on AS-related m6A regulators. The consensus clustering algorithm was employed to estimate the numbers and robustness of clusters^16^. The robustness of classification was guaranteed using the R package “ConsensusClusterPlus” with the above steps for 1000 iterations. The overall expression status of different m6A regulators related to modification patterns in AS was further validated by principal component analysis (PCA). additionally, we compared the expression status of all m6A regulators in distinct modification patterns using t-test.

### Features analysis of distinct m6A modification patterns

Distinct m6A regulators modification patterns might indicate variations in the immune status within the molecular mechanism. Hence, we proceeded to conduct a t-test to compared immunocyte subpopulations, immune reaction pathways, and HLA genes expression between two subtypes. Furthermore, we utilized the R package “limma” to perform differentially expressed analysis in order to identify disparities in gene expression between two modification patterns. Genes with a *P*-value < 0.05 were deemed significantly differently expressed genes between two modification patterns.

### Biological enrichment analysis for distinct m6A modification patterns

The functional enrichment analysis of Gene Ontology biological process (GO-BP) and Kyoto Encyclopedia of Genes and Genomes (KEGG) pathways was conducted to investigate the biological functions of the two modification patterns. The R package “clusterProfiler” was utilized for GO-BP enrichment analysis based on the differently expressed genes between two subtypes. GO-BP terms with a *P*-value < 0.05 were considered significantly enriched functional annotations. For KEGG pathway enrichment analysis^22^, we obtained the gene sets from Molecular Signatures Database (MSigDB), specifically “c2.cp.kegg.v7.5.1.symbols”. We then transformed the expression matrix of gene sets into a pathway activation score matrix using the GSVA (gene-set variation analysis) algorithm^23^. A t-test was employed to compare the pathway activation score matrix between the two modification patterns, with pathways having a *P*-value < 0.05 being considered statistically significant^24^. These results were visualized using a heatmap.

### Module analysis of m6A modification patterns

To identify the significant modules associated with different modification patterns, we employed weighted gene co-expression network analysis (WGCNA) using the R package "WGCNA"^25^. Initially, all samples were clustered to detect any potential outliers. Subsequently, the WGCNA method was used to construct a co-expression network based on the Pearson's paired correlation coefficient matrix. The dynamic tree cut algorithm was applied to cluster genes into different functional modules represented by different colors. The correlation between modules and phenotypes was evaluated through the calculation of module membership (MM) and gene significance (GS). Finally, key module gene information from the selected modules was used for further analysis.

## Results

### Identification of AS-related m6A modification regulators

**Figure 1:**
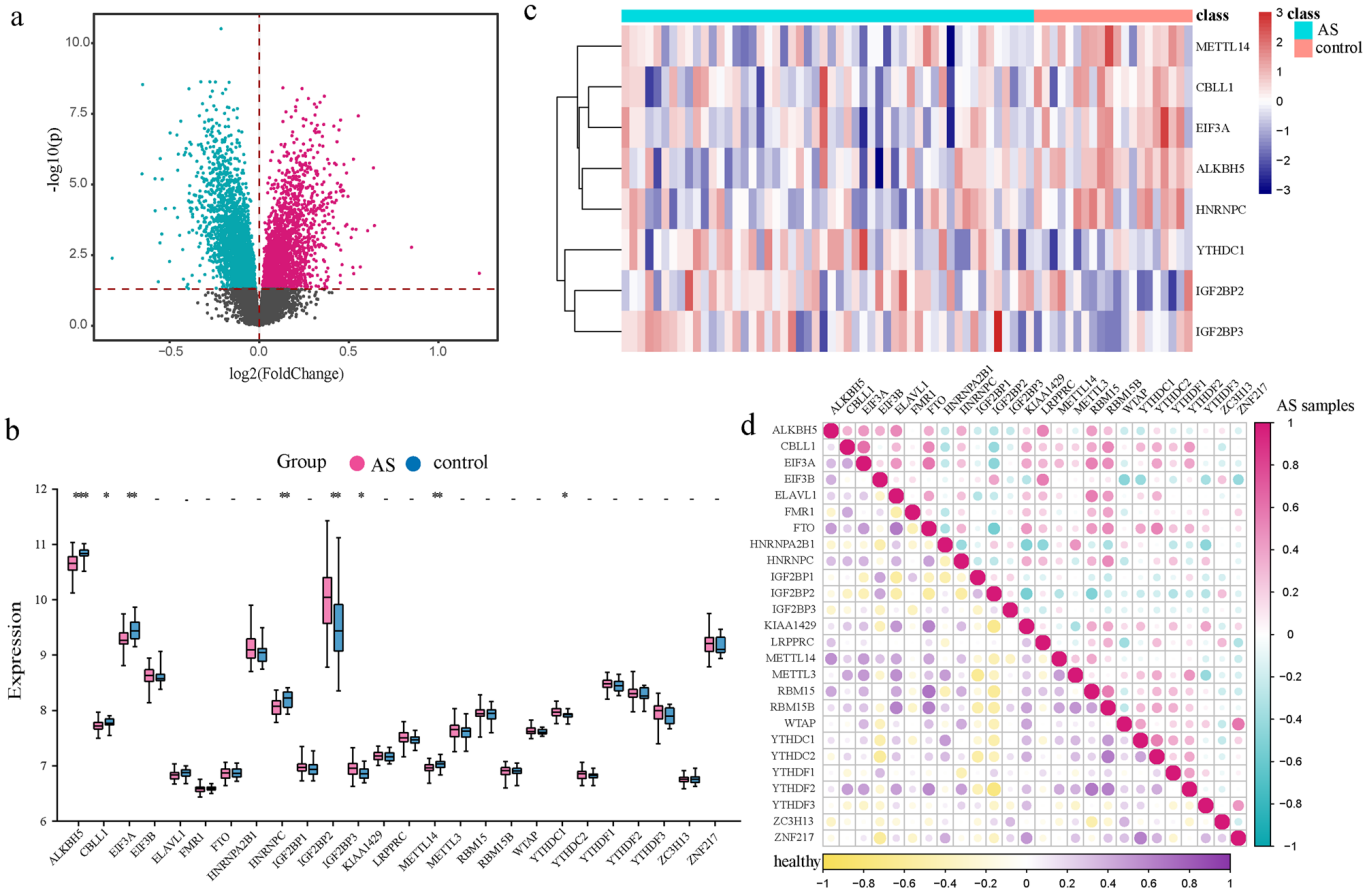
Expression landscape of m6A regulators in AS. (**a**) Volcano plot of differently expressed genes between AS samples and healthy samples. (**b**) The expression status of 26 m6A regulators. (**c**) AS-related dysregulated m6A regulators. (**d**) Comparison of all m6A regulators between AS samples and healthy samples.

In the present study, the expression status of all genes between AS samples and healthy control samples was compared through differential expression analysis. The DEGs were identified and visualized in the volcano plot (Fig. 1a). Additionally, we obtained the expression status of m6A regulators by mapping 26 m6A regulators into the GSE73754 dataset (Fig. 1b). Among these regulators, 8 DEMRGs (ALKBH5, CBLL1, EIF3A, HNRNPC, IGF2BP2, IGF2BP3, METTL14 and YTHDC1) were found between AS samples and healthy controls (Fig. 1c). These 8 DEMRGs included writer (CBLL1 and METTL14), eraser (ALKBH5) and reader (EIF3A, HNRNPC, IGF2BP2, IGF2BP3, and YTHDC1), indicating that these three types of m6A regulators might play vital roles together in the pathogenesis of AS. To further reveal the relationships among m6A regulators, we conducted correlation analysis and observed that EIF3A and CBLL1 exhibited the strongest correlation in AS samples, indicating their potential involvement in regulated function together (Fig. 1d).

### Construction of m6A related classifier to distinguish AS and healthy samples

**Figure 2:**
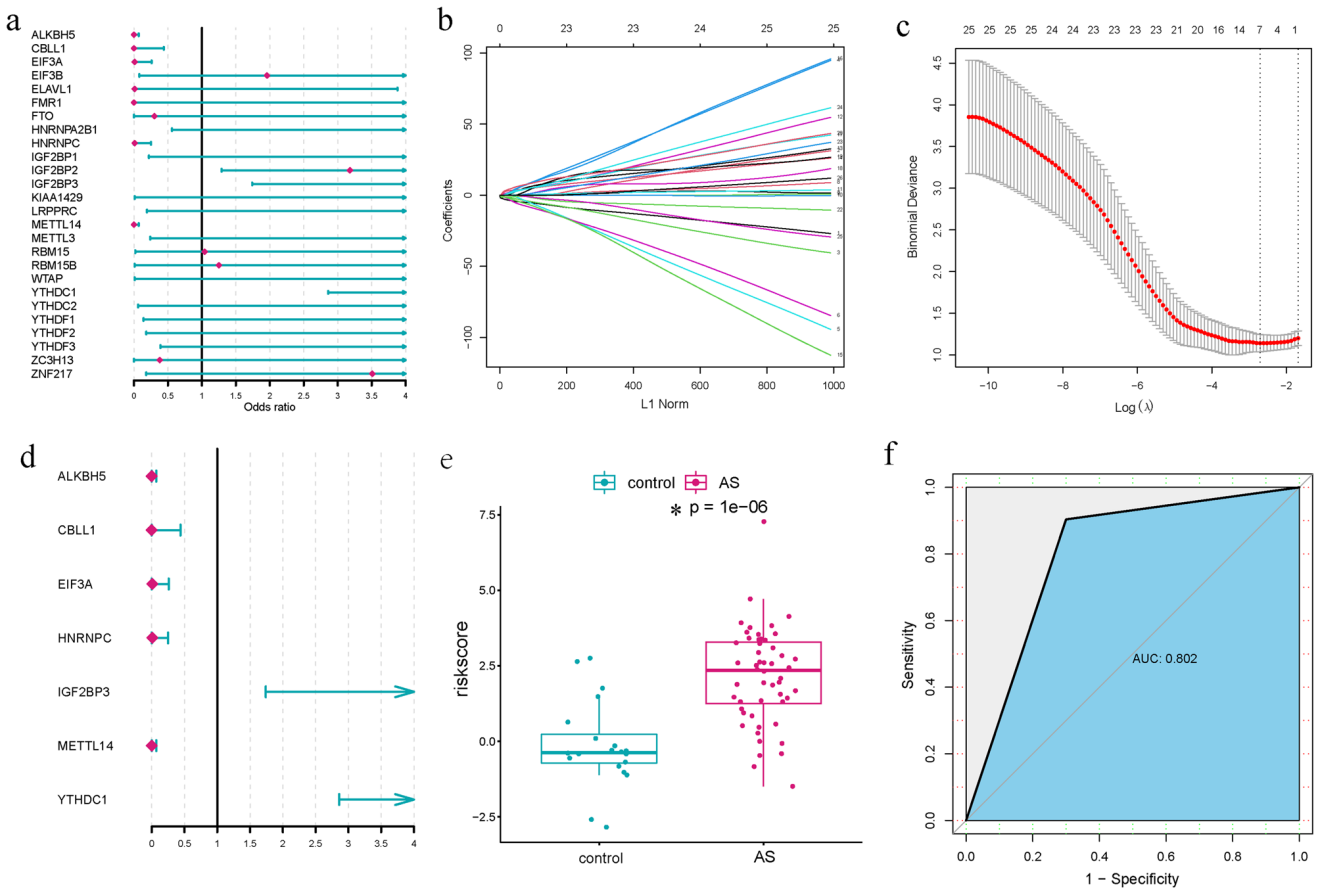
Construction of m6A related classifier. (**a**) Univariate logistic regression analysis of the relationships between 26 dysregulated m6A regulators and AS. (**b**) Least absolute shrinkage and selection operator (LASSO) coefficient profiles of dysregulated m6A regulators (*P* < 0.05). (**c**) tenfold cross-validation for tuning parameter selection in the LASSO regression. The partial likelihood deviance is plotted against log (λ), where λ is the tuning parameter. Partial likelihood deviance values are shown, with error bars representing SE. The dotted vertical lines are drawn at the optimal values by minimum criteria and 1-SE criteria. (**d**) Construction of a classifier consisting of 7 m6A regulators with multivariate logistic regression. (**e**) The risk distribution between AS and control samples, where AS has a much higher risk score than control samples. (**f**) ROC curve evaluated the discrimination ability of classifier model.

In order to explore the role of the m6A regulators in the pathogenesis of AS, we conducted a series of bioinformatic analyses to identify its crucial biological features. Firstly, univariate logistic regression analysis was utilized to examine the relationship between the 26 m6A regulators and AS (Fig. 2a). Interestingly, 8 m6A regulators (*P* < 0.05) were found to be significantly associated with AS, consistent with DEMRGs, indicating that their important roles in AS. Next, LASSO regression was employed for feature selection and dimensionality reduction resulting in the recognition of 7 essential m6A regulators (ALKBH5, CBLL1, EIF3A, HNRNPC, IGF2BP3, METTL14 and YTHDC1) for AS (Fig. 2b,c). Subsequently, multivariate logistic regression was conducted to develop a classifier that could distinguish between AS and healthy samples (Fig. 2d). Risk scores were calculated for each of samples, and the results demonstrated that the m6A related classifier composed of the 7 selected m6A regulators effectively differentiated healthy samples from AS samples (Fig. 2e), with significantly higher risk score observed in AS samples compared to healthy samples (*P* < 0.0001). Finally, ROC analysis was conducted to evaluate the recognition ability of the classifier, yielding an area under the curve was 0.802, indicating excellent discrimination ability in distinguishing AS from healthy subjects (Fig. 2f). Additionally, we verified the performance of the m6A-related classifier using the GSE25101 dataset, which yielded similar results, suggesting its robustness (Supplementary Fig. S1).

### Validation of differentially expressed m6A modification regulators

**Figure 3:**
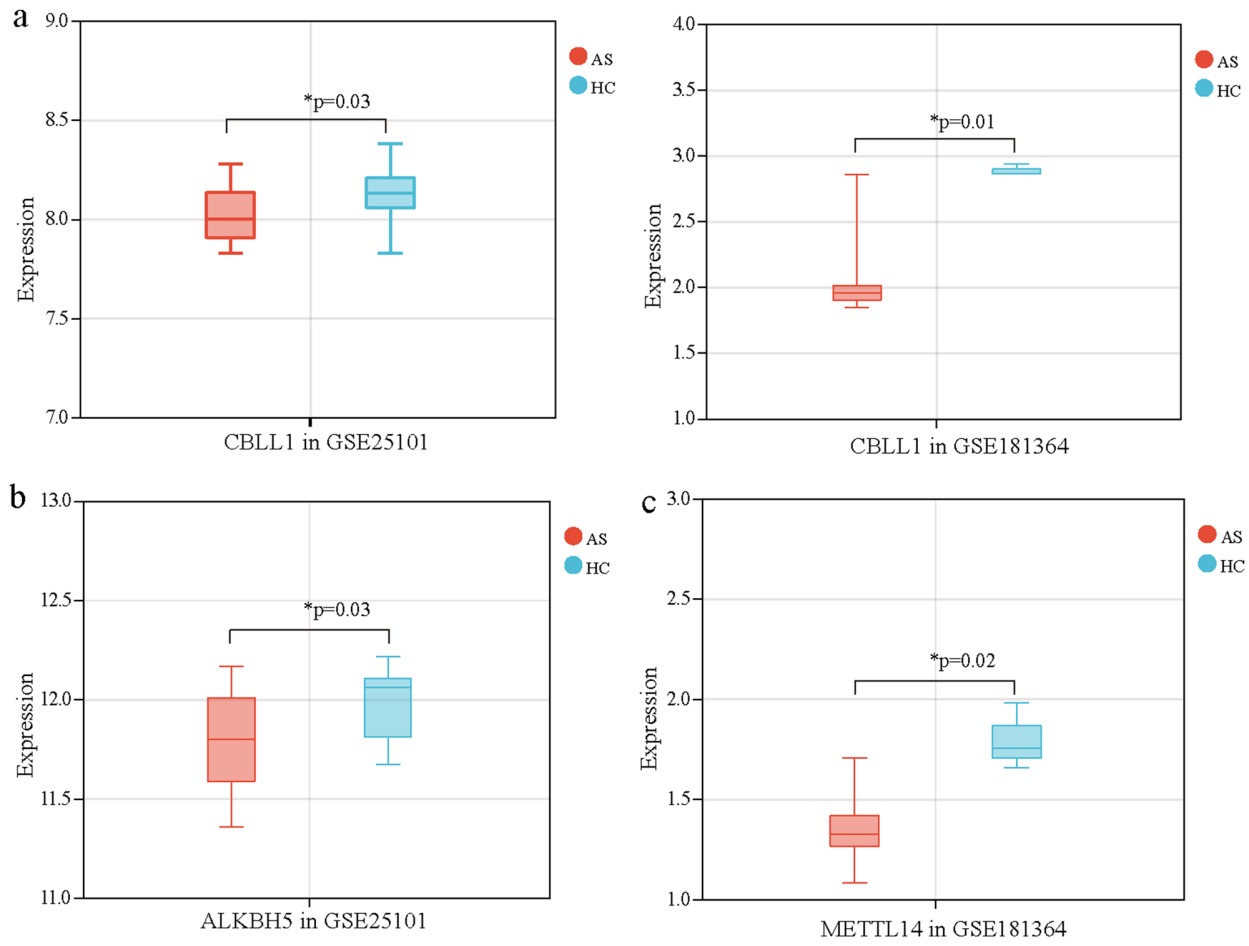
Validation of differently expressed m6A modification regulators. (**a**) The expression of CBLL1 in GSE25101 dataset and GSE181364 dataset. (**b**) The expression of ALKBH5 in GSE25101 dataset. (**c**) The expression of METTL14 in GSE181364 dataset.

The expression status of m6A modification regulators were validated using two additional datasets (GSE25101 and GSE181364). As a result, 8 out of 26 m6A regulators exhibited differentially expressed in GSE25101 dataset (Supplementary Fig. S5) and 7 were differentially expressed in GSE181364 dataset (Supplementary Fig. S6). Among them, CBLL1 was downregulated in both datasets (Fig. 3a), ALKBH5 was downregulated in GSE25101 dataset (Fig. 3b), and METTL14 was downregulated in GSE181364 dataset (Fig. 3c). These findings were consistent with GSE73754 dataset, suggesting that CBLL1, ALKBH5, and METTL14 may be particularly important m6A regulators in the pathogenesis of AS.

### The correlation between m6A regulators and immune characteristics of AS

**Figure 4:**
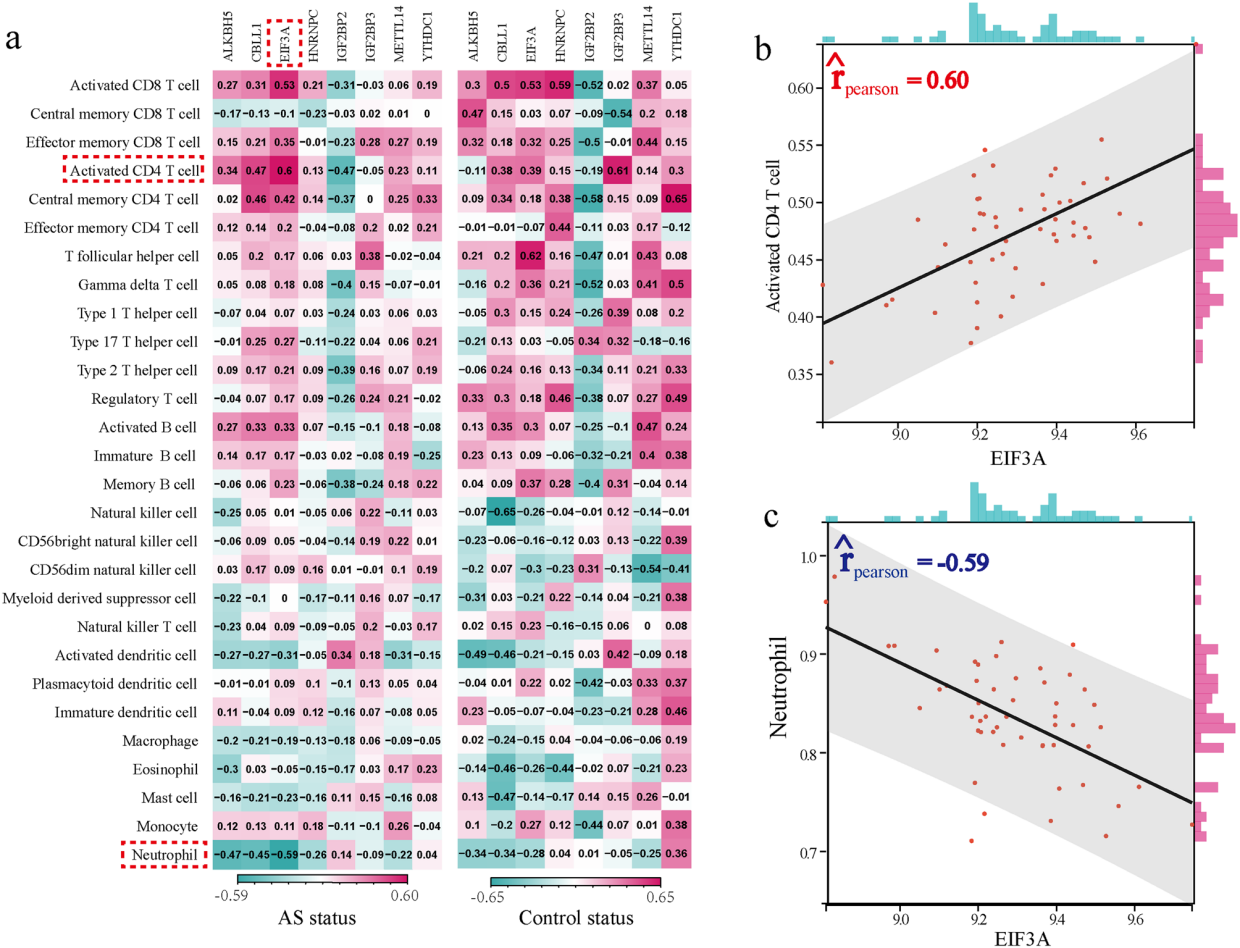
The correlation between immune cell subpopulations and dysregulated m6A regulators. (**a**) The dot-plot showed the correlations between each immunocyte cell type and each dysregulated m6A regulators in AS samples and healthy samples. (**b**) The most positively correlated immunocyte-m6A regulators pair in AS. (**c**) The most negatively correlated immunocyte-m6A regulators in AS.

**Figure 5:**
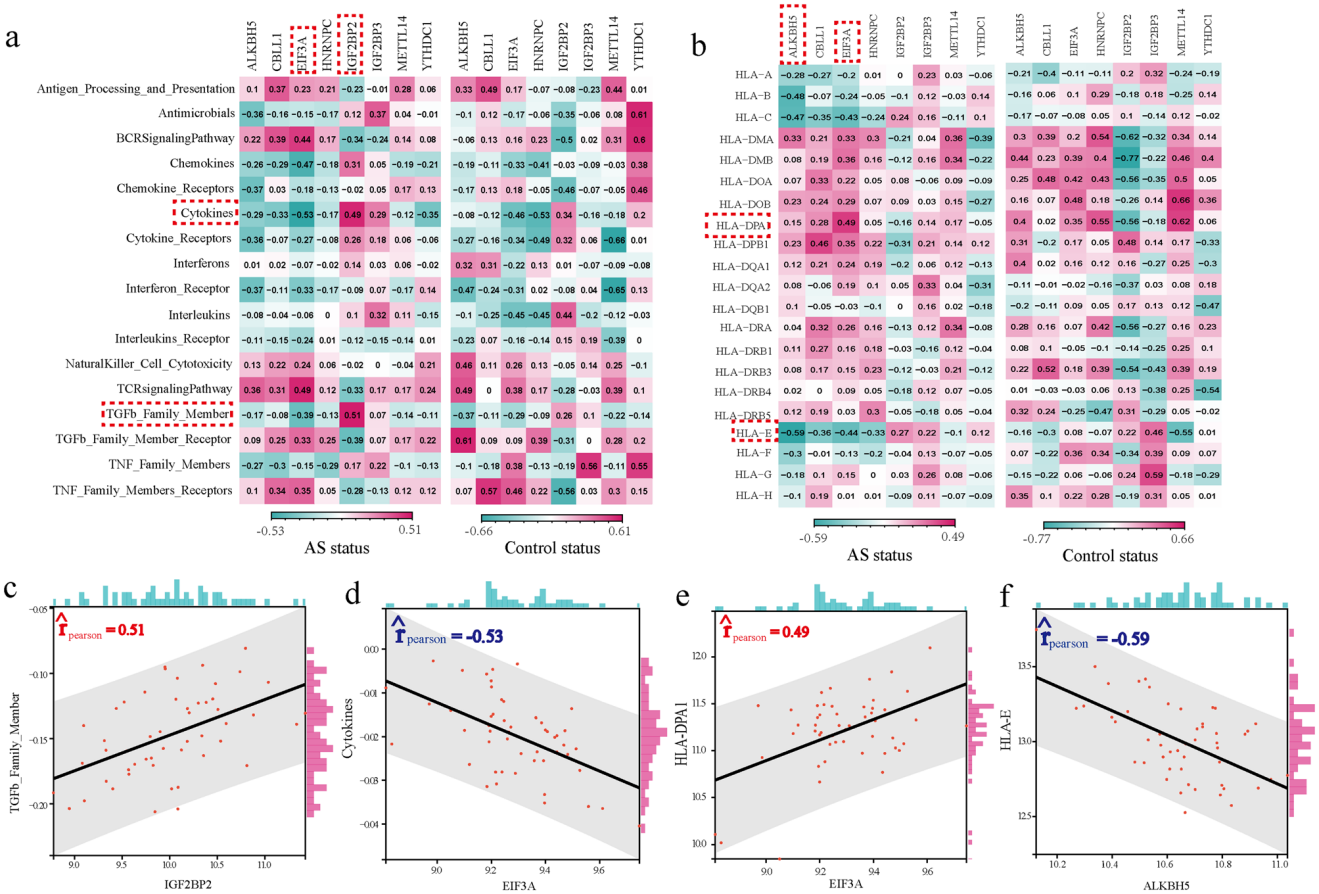
The correlation between immune reaction pathways and HLA genes and dysregulated m6A regulators. (**a**) The dot-plot showed the correlations between each immune reaction pathway and each dysregulated m6A regulators in AS samples and healthy samples. (**b**) The dot-plot showed the correlations between each HLA gene and each dysregulated m6A regulators in AS samples and healthy samples. (**c**) The most positively correlated immune reaction pathway-m6A regulators pair in AS. (**d**) The most negatively correlated immune reaction pathway-m6A regulators in AS. (**e**) The most positively correlated HLA-m6A regulators pair in AS. (**f**) The most negatively correlated HLA-m6A regulators in AS.

To investigate the correlations between AS-related m6A regulators and immune characteristics, we employed correlation analysis for m6A regulators with immunocytes, immune reaction pathways, and HLA gene expression^26^. The enrichment abundance of 28 immunocyte subpopulations between AS and healthy samples were calculated by the ssGSEA algorithm (Supplementary Fig. S2). We observed significant changes in the infiltrating immunocyte fractions in AS, with several subpopulations being upregulated (e.g., Central memory CD8 T cell, Effector memory CD4 T cell, CD56 bright natural killer cell) and others being downregulated (e.g., Activated CD8 T cell, Type 1 T helper cell). Additionally, the correlation analysis between AS-related m6A regulators and immunocyte subpopulations revealed alterations in the correlations between these regulators and immune cell subpopulations in AS compared to healthy status (Fig. 4a). Notably, EIF3A exhibited the strongest positive correlation with Activated CD4 T cell and the strongest negative correlation with Neutrophil (Fig. 4b,c), suggesting that lower expression of EIF3A was associated with reduced Activated CD4 T cell levels and increased neutrophil levels in AS. Similarly, the activity of immune reaction pathways and the expression levels of HLA genes were also analyzed (Supplementary Figs. S3, S4). Significant changes in immune reaction pathways and HLA gene expression were observed between AS and healthy samples, such as increased activity of Interferon Receptor and elevated expression of HLA-B and HLA-C in AS samples. Furthermore, we explored the correlations between immune reaction pathways/HLA gene expression and m6A regulators (Fig. 5a, b). Notably, IGF2BP2-TGF-β family member exhibited the strongest positive correlation among immune reaction pathways, while EIF3A-cytokines showed the strongest negative correlation (Fig. 5c,d). Regarding HLA gene expression, EIF3A-HLA-DPA1 demonstrated the strongest positive correlation, while ALKBH5-HLA-E exhibited the strongest negative correlation (Fig. 5e,f).

### Identification of distinct modification pattern mediated by m6A regulators in AS

**Figure 6:**
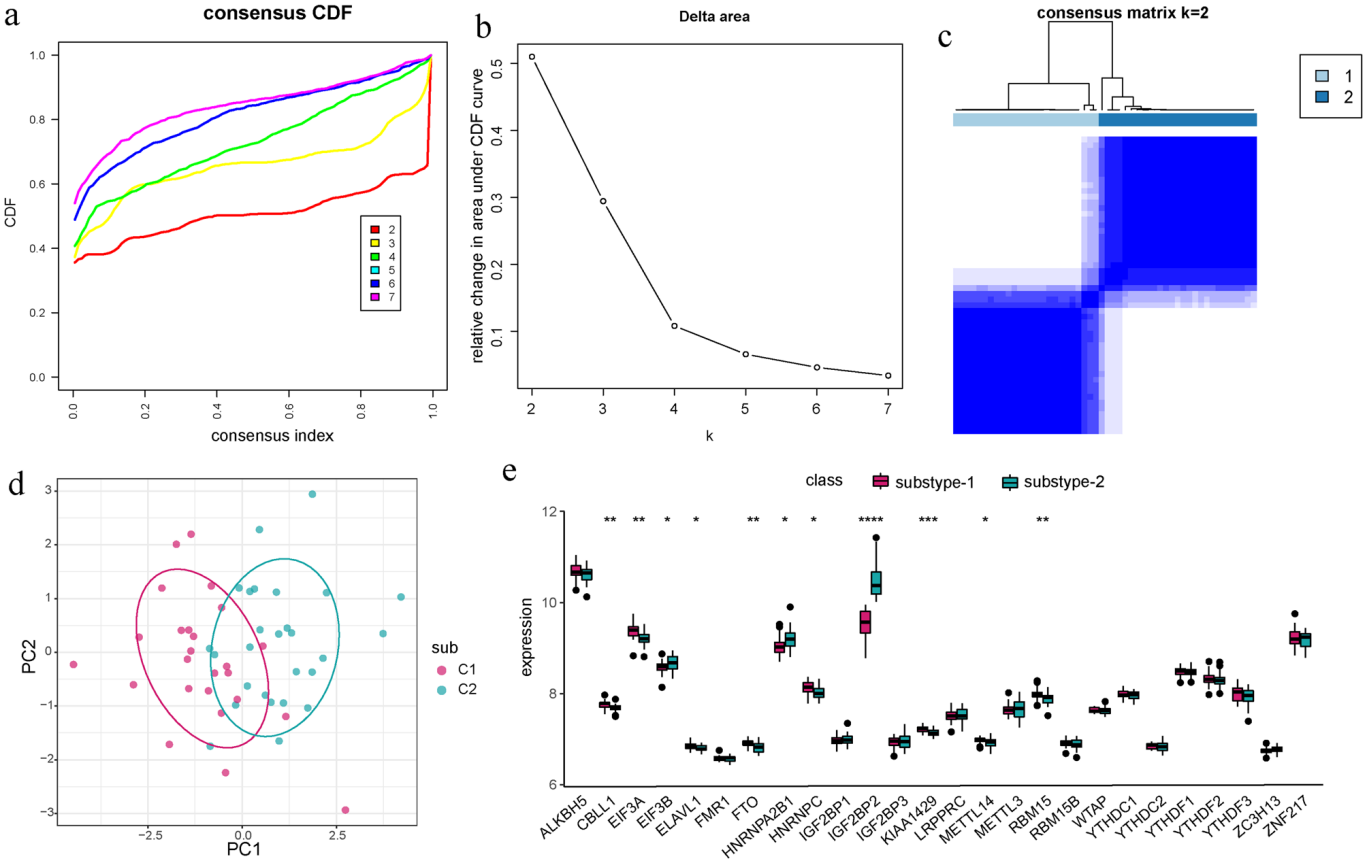
Recognition of distinct m6A modification patterns based on unsupervised consensus clustering. (**a**) Consensus clustering cumulative distribution function (CDF) for k = 2–7. (**b**) Relative change in area under CDF curve for k = 2–7. (**c**) Heatmap of the matrix of co-occurrence proportions for AS samples. (**d**) PCA analysis for the transcriptome profiles of the two different modification subtypes. (**e**) The expression status of all m6A regulators in the two subtypes.

To explore distinct m6A modification patterns, we performed unsupervised consensus cluster analysis on AS samples based on the expression of AS-related m6A regulators using the Consensus Cluster Plus R package (Fig. 6a–c). The results revealed two subtypes: subtype-1 (n = 25) and subtype-2 (n = 27). PCA analysis showed a significant difference in transcriptome expression between the two modification patterns (Fig. 6d). Additionally, we analyzed the expression patterns of the 26 m6A regulators in the two subtypes. We found that CBLL1, EIF3A, ELAVL1, FTO, HNRNPC, KIAA1429, METTL14, and RBM15 were upregulated in subtype-1, while EIF3B, HNRNPA2B1, and IGF2BP2 were upregulated in subtype-2 (Fig. 6e), confirming the existence of diverse m6A modification patterns in AS.

### Immune characteristics in the two distinct m6A modification patterns

**Figure 7:**
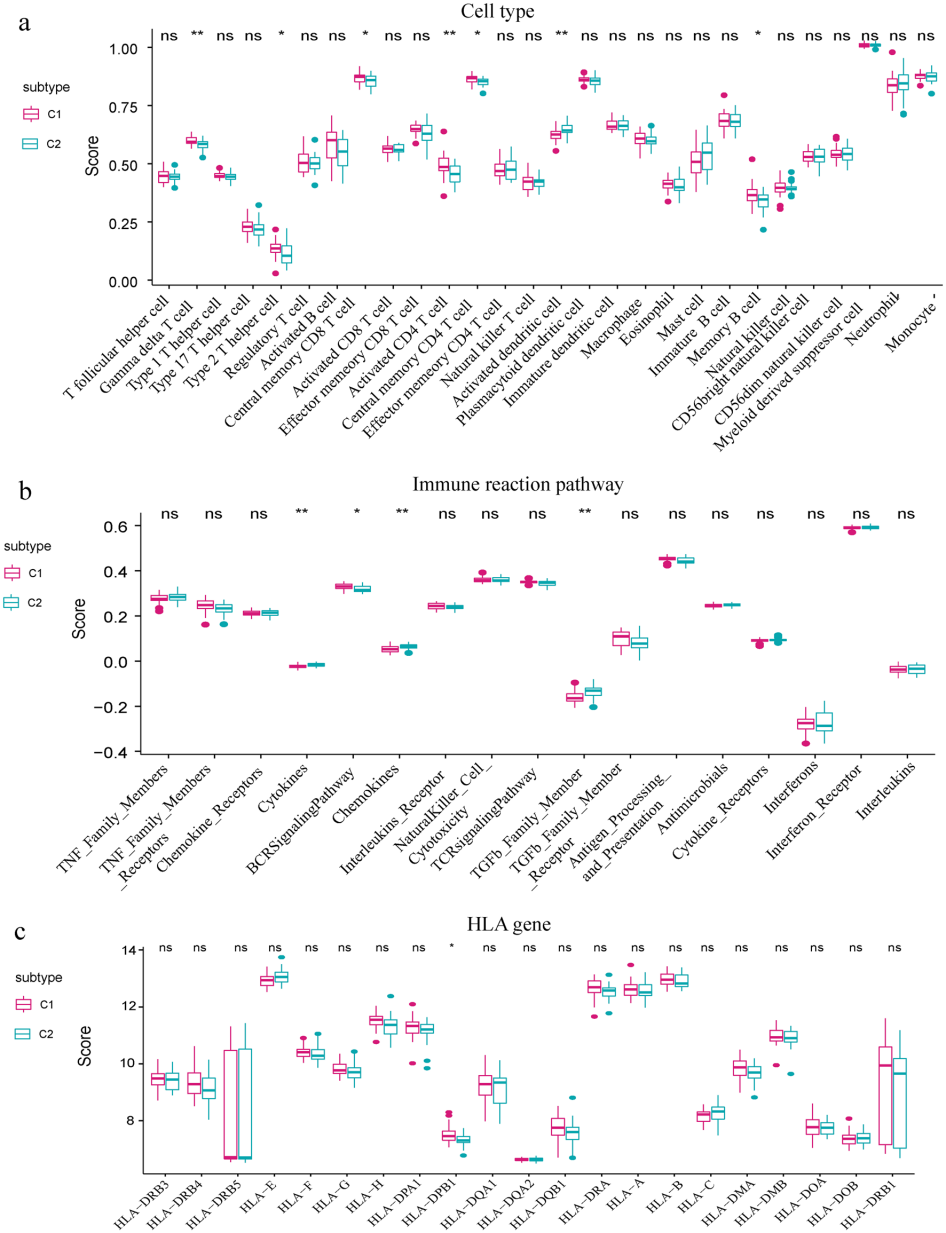
Different immune characteristics of two distinct m6A modification patterns. (**a**) The abundance differences of each immune immunocyte in two m6A modification subtypes. (**b**) The activity differences of each immune reaction in two m6A modification subtypes. (**c**) The expression differences of each HLA gene in two m6A modification subtypes.

To describe the immune characteristics of the two different m6A modification patterns, we compared the expression status of immunocyte subpopulations, immune reaction pathways, and HLA gene expression. As expected, several immune characteristics differed between the two patterns. For instance, a higher level of Gamma delta T cell, Type 2 T helper cell, Central memory CD8 T cell, Activated CD4 T cell, Central memory CD4 T cell, and Memory B cell was enriched in subtype-1, while a higher level of infiltrated Activated dendritic cell was observed in subtype-2 (Fig. 7a). In terms of immune reaction pathways, subtype-2 exhibited more active immune reaction pathways compared to subtype-1, with enrichments in Cytokines, Chemokines, and TGF-β family member pathways, while only the BCR Signaling Pathway was more active in subtype-1 (Fig. 7b). Furthermore, slight differences in HLA gene expression were observed between the two modification patterns, such as higher expression of HLA-DPA1 in subtype-1 (Fig. 7c). These findings suggest that subtype-1 is characterized by a higher number of infiltrating immunocytes and higher HLA gene expression, while subtype-2 is involved in more active immune reaction pathways. Therefore, different subtypes of m6A methylation have important regulatory effects on the formation of different immune microenvironments of AS.

### Identification of biological functions and modules of two m6A modification patterns

**Figure 8:**
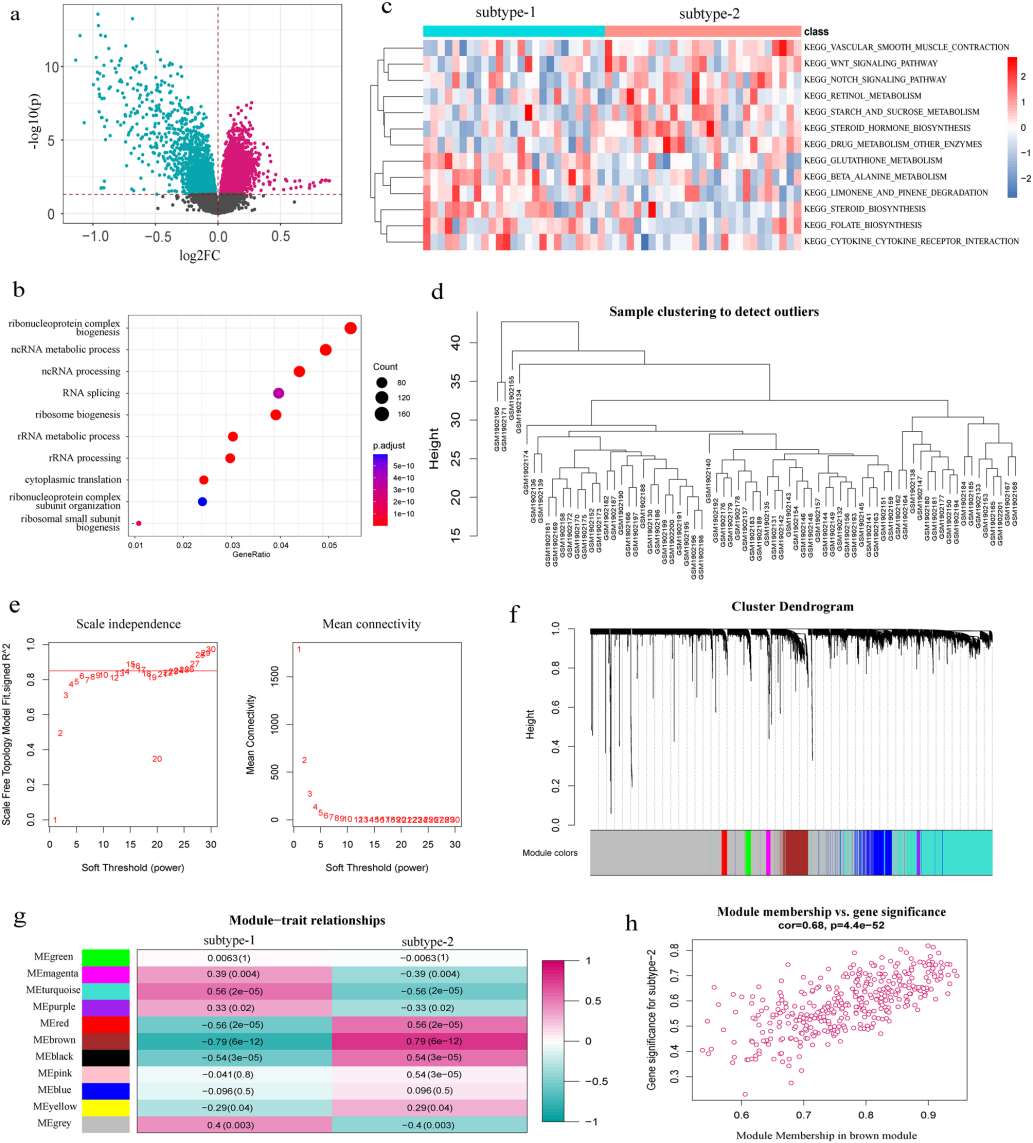
Potential biological function characteristics of different m6A modification subtypes. (**a**) The differently expressed genes between two subtypes. (**b**) The top 10 of GO-BP enrichment analysis of the above differently expressed genes. (**c**) The expression status of 13 KEGG pathways in subtype-1 and subtype-2. (**d**) The sample clustering was performed according to the expression data of all samples. The top 25% variation genes were used for the analysis by WGCNA. (**e**) Analysis of the scale free ft index and analysis of the mean connectivity for various soft-thresholding powers. (**f**) Gene dendrogram obtained by average linkage hierarchical clustering. The color row underneath the dendrogram shows the module assignment determined by the Dynamic Tree Cut, in which 11 modules were identified. (**g**) The heatmap showed the correlation between module eigengenes and the two modification patterns; WGCNA algorithm uses PCA to compute the principal components of the module matrix and defines PC1 as eigengenes. (**h**) A scatterplot of gene significance (GS) for subtype-2 versus module membership (MM) in the brown module. GS and MM exhibit a very significant correlation, implying that hub genes of the brown module also tend to be highly correlated with subtype-2. MM represented the correlation between each gene in brown module and brown module, GS represented the significance of gene in brown module and subtype-2.

To investigate the potential biological functions of the two m6A modification patterns, we first identified 5889 differently expressed genes between the two patterns (subtype-1 and subtype-2) through limma package (Fig. 8a). Subsequently, we conducted GO-BP enrichment analysis using the above differently expressed genes and found that the significantly enriched GO-BP terms primarily included ribonucleoprotein complex biogenesis, ncRNA metabolic process, and ncRNA processing (Fig. 8b). Additionally, we evaluated the activation status of KEGG pathways between subtype-1 and subtype-2 using GSVA enrichment analysis. And 13 significantly dysregulated pathways (*P* < 0.05) were identified (Fig. 8c). For example, glutathione metabolism and beta alanine metabolism pathways were mainly enriched in subtype-1, while steroid hormone biosynthesis, vascular smooth muscle contraction, and notch signaling pathway were mainly enriched in subtype-2. Furthermore, we employed the WGCNA algorithm to construct a comprehensive gene landscape and identify gene–gene modules. Sample clustering was based on the expression data from all samples, with the top 25% variation genes used for the analysis by WGCNA. A gene dendrogram was obtained through average linkage hierarchical clustering, with the module assignment determined by the Dynamic Tree Cut (Fig. 8d–f). Finally, we identified 11 gene modules (including green, magenta, turquoise, purple, red, brown, black, pink, blue, yellow, and grey) and further analyzed their connection with the two m6A modification patterns (subtype-1 and subtype-2). Interestingly, we observed that the brown module exhibited the highest positive correlation with subtype-2 (Fig. 8g), indicating a close relationship between the genes in the brown module and subtype-2 (Fig. 8h).

## Discussion

AS is a complex and potentially debilitating autoimmune disease with an insidious onset that can develop into radiological sacroiliitis over a number of years. With the continuous improvement of diagnostic technology of AS, an increasing number of AS patients were diagnosed. It is widely known that HLA-B27 is the main genetic factor predisposing to AS, present in about 95% of patients^27^. However, only about 5% of the general population positive for HLA-B27 will develop AS, and HLA-B27 accounts for less than 50% of the total risk^28^. This suggests that other susceptibility genes are involved in the pathogenesis of AS. Therefore, it is indispensable to explore the pathogenic factors associated with AS and determine diagnostic markers. m6A methylation modification is a novel epigenetic modification of gene expression regulation involved in numerous biological processes. Several researches have shown that m6A modifications are implicated in innate and adaptive immune responses^11^. In recent years, researchers have discovered that m6A modifications play a crucial part in various autoimmune diseases^29^. However, few studies have focused on the roles of m6A modification in the development of AS. Therefore, we conducted a systematic investigation of m6A modification patterns in the immune microenvironment of AS and explored the immune characteristics associated with m6A modification.

In this study, we identified 8 differentially expressed m6A regulators between AS and healthy samples, including 5 readers (EIF3A, HNRNPC, IGF2BP2, IGF2BP3, and YTHDC1), two writers (CBLL1 and METTL14), and one eraser (ALKBH5). Correlation analysis revealed that EIF3A and CBLL1 exhibited the strongest correlation among m6A regulators in AS samples. Previous studies have indicated that EIF3A can regulate cell response to DNA damage through mTOR signaling^30^, which is a significant pathway in the pathogenesis of AS^31^, suggesting that EIF3A may play a crucial role in AS pathogenesis. CBLL1 is known to have pleiotropic effects on inducing or inhibiting osteoclast activity^32^ and the role of CBLL1 in AS needs to be further explored. To assess the recognition ability of m6A regulators in AS samples, we performed univariate logistic regression analysis, LASSO regression analysis, and multivariate regression analysis. As a result, we constructed a classifier composed of 7 AS-related m6A regulators. The ROC curve demonstrated excellent recognition ability of the classifier. Importantly, the classifier showed robust performance in the validation dataset (GSE25101). Furthermore, we explored immune characteristics associated with m6A regulators in AS using the ssGSEA algorithm to evaluate immune cell abundance, immune response activity, and HLA gene expression. We observed an upregulation of Neutrophils in AS samples, which have been implicated in the immune inflammatory response of AS^33^. Neutrophils might have a buffering function in AS, releasing pre-stored pro-inflammatory cytokines into already inflamed tissue^34^. HLA gene expression results demonstrated increased expression of HLA-B and HLA-C in AS samples. HLA-B27 allele is a major risk factor for the development of AS and a crucial biomarker of the disease. Kim. et al. reported HLA-C amino-acid variant in addition to HLA-B27 confers risk for ankylosing spondylitis in the Korean population^35^.

Additionally, we analyzed the correlation between m6A regulators and immune characteristics of AS. The results showed that the most negatively correlated m6A regulator-immune cell subpopulation pair was EIF3A-Neutrophil. In our study, EIF3A was significantly down-regulated in AS, while data from a total of 9707 routine blood tests showed that the neutrophil count was significantly higher in AS patients than in controls^36^, which provided support for our result. In the immune reaction pathways, IGF2BP2 showed the strongest positive correlation with a TGF-β family member. Moreover, a previous study indicated that high levels of active TGF-β induced new bone formation in the ligaments and facilitated the progression of AS^37^. TGF-β1 is increased in the ligamentum flava and paraspinal muscle tissues of AS patients, and it may be the most important morphogenetic factor mediating the differentiation of pathological fibroblasts into myofibroblasts^38^. These m6A regulators are closely related to these immune characteristics, illustrating that m6A modification plays a significant role in the regulation of the AS immune microenvironment.

Given the heterogeneity of the disease, we used the m6A regulator expression profiles for consensus clustering of AS to identify different modification patterns and further explored the immune characteristics of each pattern. We found that CBLL1, ELAVL1, FTO, HNRNPC, KIAA1429, METTL14 and RBM15 were overexpressed in subtype-1, whereas EIF3A, EIF3B, HNRNPA2B1 and IGF2BP2 were overexpressed in subtype-2. Subtype-1 had more immune cell infiltration compared to subtype-2. Subtype-2 exhibited more active immune reactions than subtype-1 in terms of immune reaction pathways. Additionally, we identified the biological functions and modules of the two m6A modification patterns. Comparing the two patterns, we found that subtype-2 had more activation in famous notch signaling pathway. It has been reported that overexpression of ligands or receptors of the Notch signaling pathway may be potentially harmful to osteoclasts and osteoblast precursors, affecting cell differentiation and playing a role in bone development^39^, which may contribute to ligament ossification of hip joints in AS patient^40^. Furtherly, the results of WGCNA analysis showed that brown module was the most positively correlated with subtype-2 and the most negatively correlated with subtype-1. This classification strategy of immune subtypes can help us understand the underlying mechanisms of immune regulation so that precise therapies can be applied, and AS can be classified at the molecular or immune level. A recent study used this approach to identify two distinct patterns of m6A modification in myasthenia gravis. The results enhance our understanding of autoimmune diseases and help us develop more effective immunotherapy strategies^41^. In conclusion, this subtype classification strategy contributes to explain the heterogeneity of the disease, and provides a theoretical basis for realizing the potential molecular mechanism of AS and formulating better individualized treatment plans.

Finally, we validated the expression of m6A modification regulators using extra two datasets. However, the results suggested only CBLL1 (Cbl Proto-Oncogene-Like 1, writer), ALKBH5 (AlkB homolog 5, eraser), and METTL14 (methyltransferase-like 14, writer) dysregulated in the other two datasets. This may due to differences in the time of sample collection or the effect of the treatment on the disease. Chen et al. reported that the loss of METTL14 decreased the expression of FOXO3a (Forkhead box O3a), leading to the damage of autophagic flux and the aggravation of inflammation in AS^42^. The research manifested that the mRNA expressions ofALKBH5 in PBMC were decreased in patients with AS and low expression of ALKBH5 can enhance the osteogenesis of MSCs through activation of the PI3K/AKT pathway^43^. CBLL1 possessed critical pleiotropic effects on induction or inhibition of osteoclast activity^32^. We will further investigate the role of these three m6A regulators in the pathogenesis of AS in the future.

This study systematically analyzed the relationship between the m6A regulator modification and the immune microenvironment of AS for the first time. These results provide a new direction for studying the immune-related AS pathogenesis from the perspective of m6A modification mechanism. However, there are some limitations to the study. Firstly, this study is based on bioinformatics analysis, and many results are theoretically valid without experimental verification. Therefore, our research results need to be verified by subsequent experiments. Secondly, the analysis of immune cells is also the most widely used method to quantify the number of immune cells, but single-cell sequencing is still needed to obtain the most accurate number of immune cells. In addition, we cannot obtain some data, such AS serum test results and clinical features, so it is difficult to reveal the important role of m6A in AS immune regulation from multiple dimensions to a large extent, and the analysis results are relatively simple. Nevertheless, our study does reveal the important influence of m6A modification on the molecular mechanism of AS, and provides a new perspective for understanding the underlying pathogenesis of AS.

## Conclusion

In summary, our work reveals the potential mechanism by which m6A regulator modification affects the immune properties of AS. The analysis on the expression pattern of m6A regulators provides a new perspective for understanding the pathogenesis of AS and opens up a new direction for researchers to explore the molecular mechanism of other diseases.

### Supplementary Information

Supplementary Figure S1.

Supplementary Figure S2.

Supplementary Figure S3.

Supplementary Figure S4.

Supplementary Figure S5.

Supplementary Figure S6.

Supplementary Legends.

Supplementary Table S1.

## Data Availability

Data used for analysis can be found at GEO Accession (GSE73754, GSE25101, and GSE181364). The other datasets used/generated in this study are available from the corresponding authors on reasonable request.
