# Supplementary figures and images for: Analysis of m6A regulators related immune characteristics in ankylosing spondylitis by integrated bioinformatics and computational strategies
Source: Sci Rep. 2024 Feb 1;14:2724. doi: 10.1038/s41598-024-53184-z (PMC10834589; doi:10.1038/s41598-024-53184-z)

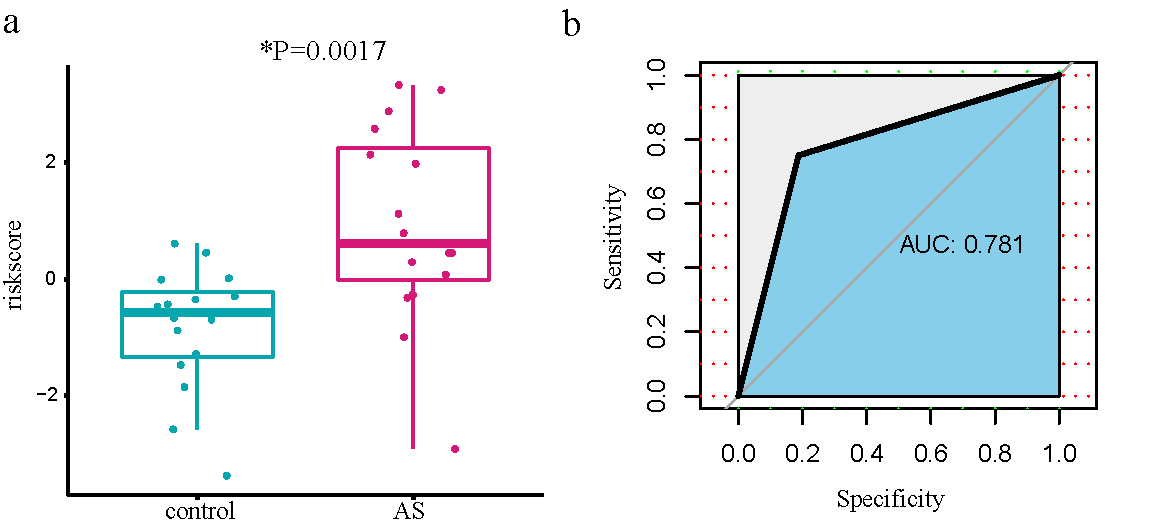

Supplement: Supplementary file 1 — Supplementary Figure S1. [file 41598_2024_53184_MOESM1_ESM.tif]

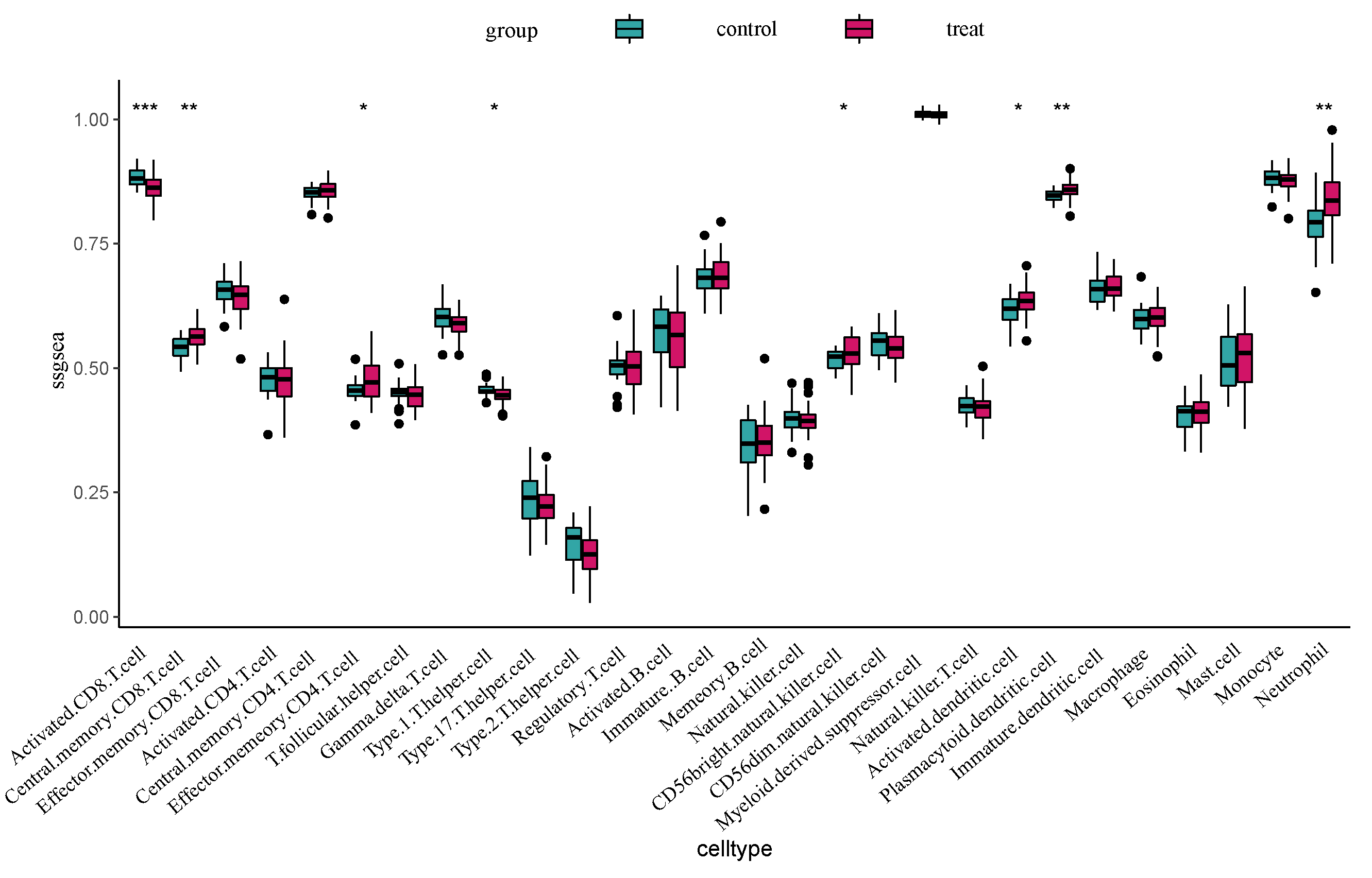

Supplement: Supplementary file 2 — Supplementary Figure S2. [file 41598_2024_53184_MOESM2_ESM.tif]

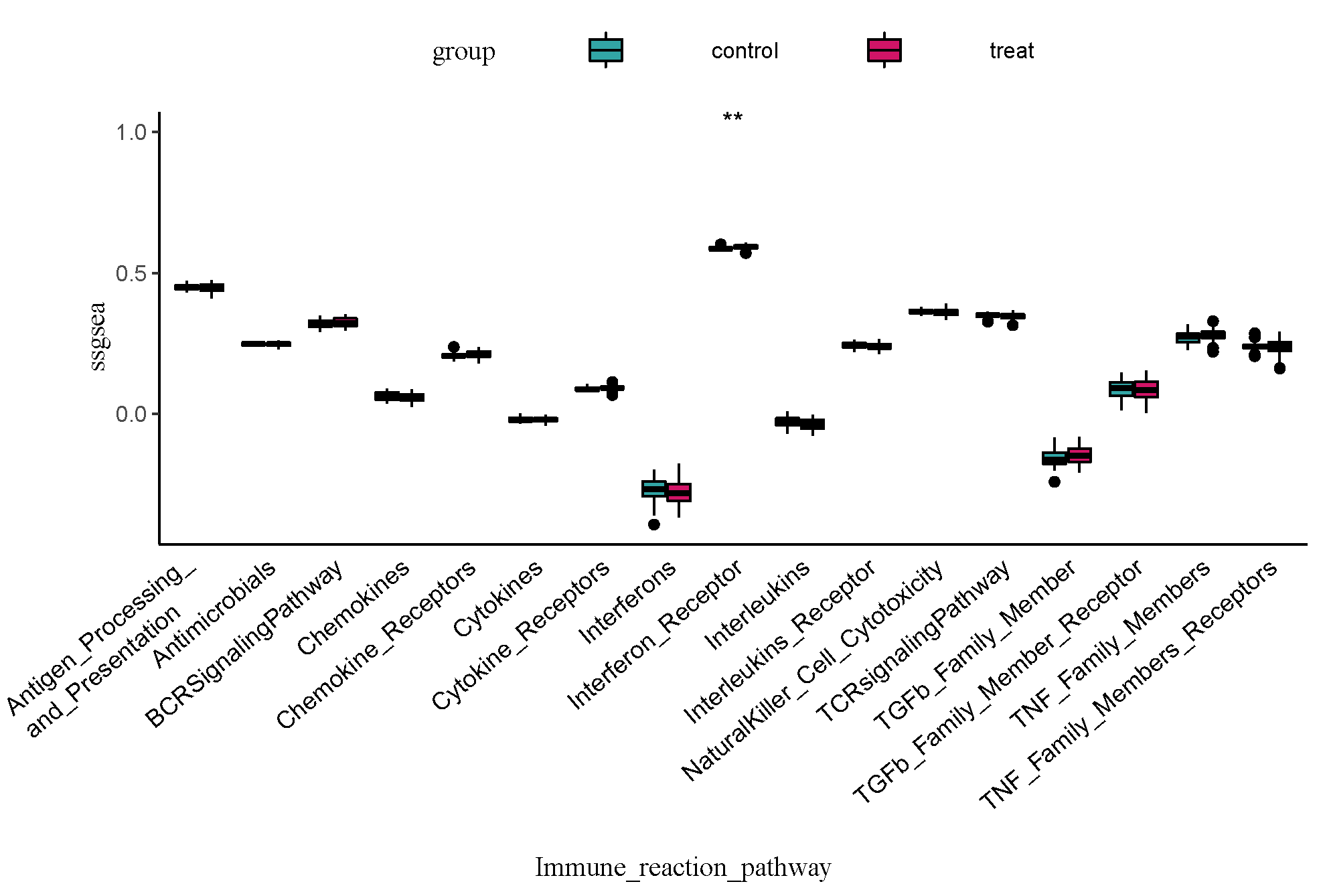

Supplement: Supplementary file 3 — Supplementary Figure S3. [file 41598_2024_53184_MOESM3_ESM.tif]

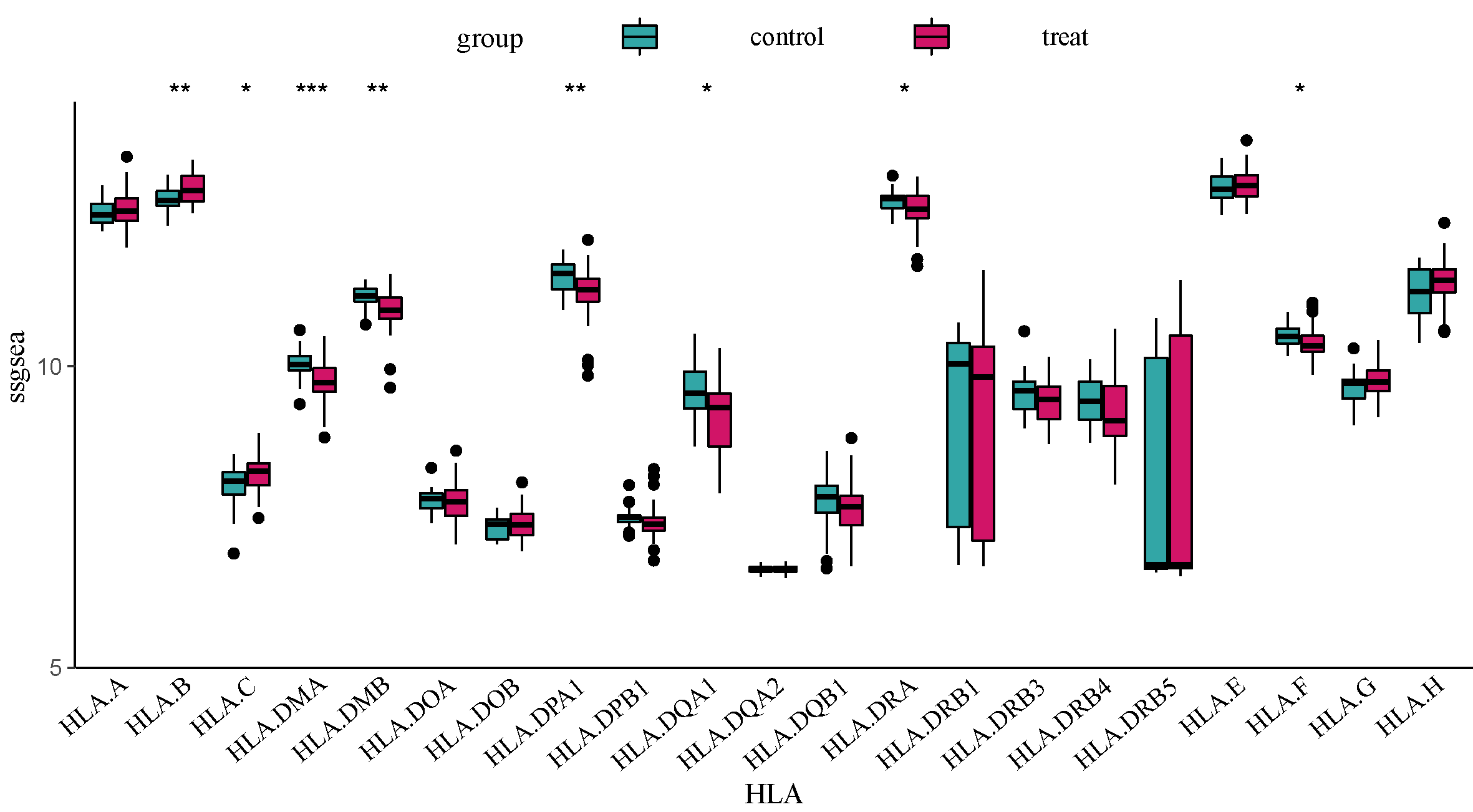

Supplement: Supplementary file 4 — Supplementary Figure S4. [file 41598_2024_53184_MOESM4_ESM.tif]

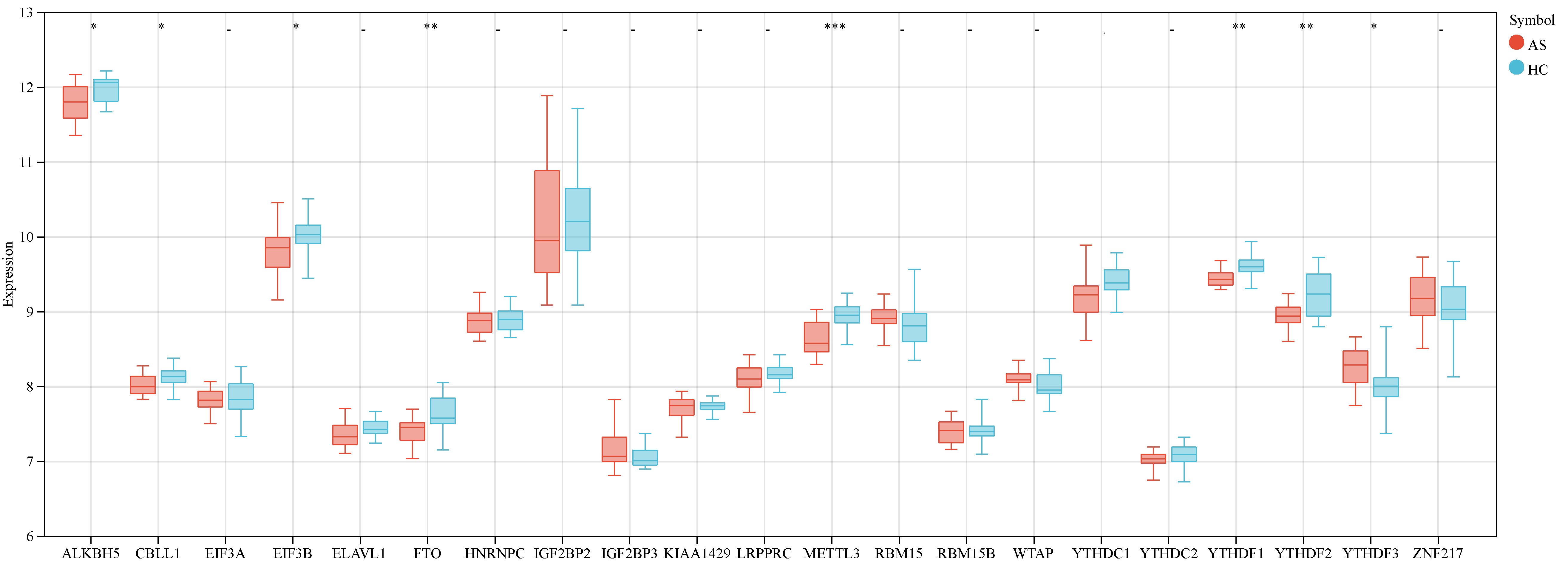

Supplement: Supplementary file 5 — Supplementary Figure S5. [file 41598_2024_53184_MOESM5_ESM.tif]

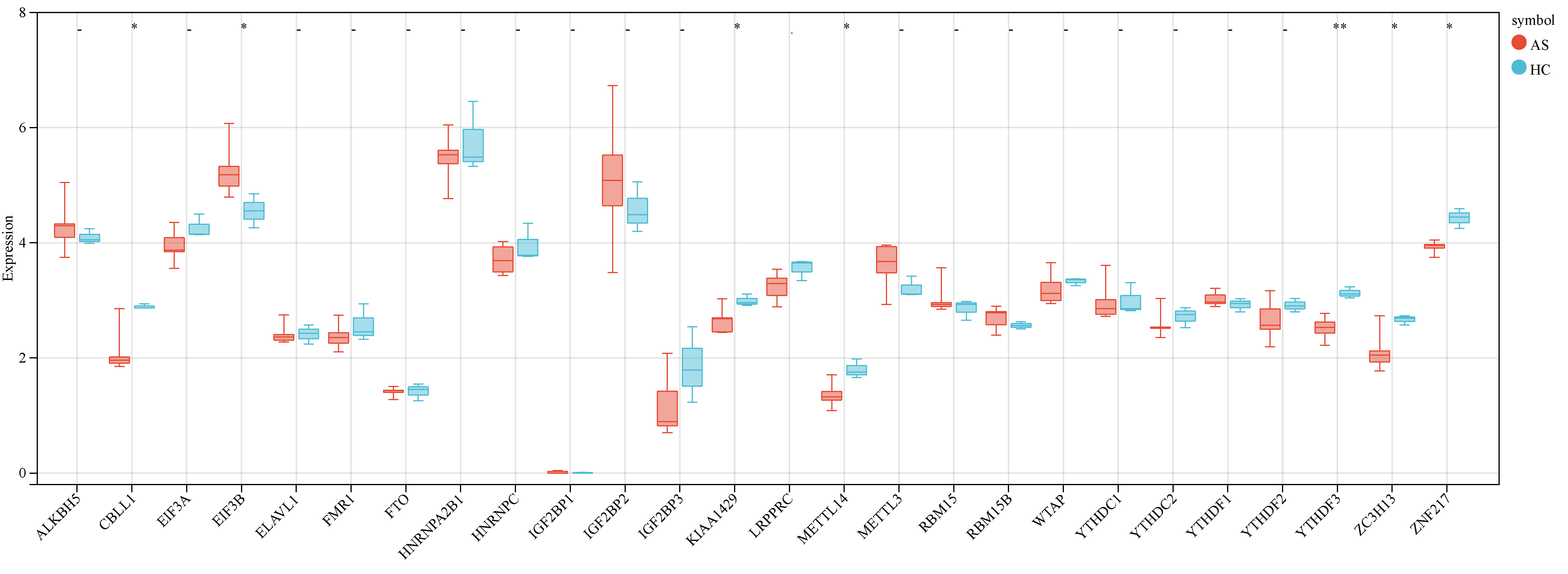

Supplement: Supplementary file 6 — Supplementary Figure S6. [file 41598_2024_53184_MOESM6_ESM.tif]
