# Supplementary material for: Analysis of m6A regulators related immune characteristics in ankylosing spondylitis by integrated bioinformatics and computational strategies
Source: Sci Rep. 2024 Feb 1;14:2724. doi: 10.1038/s41598-024-53184-z (PMC10834589; doi:10.1038/s41598-024-53184-z)
Supplement: Supplementary file 7 — Supplementary Legends. [file 41598_2024_53184_MOESM7_ESM.docx]

**Supplementary** **Figure 1** The validation of m6A related classifier discrimination ability using GSE25101 dataset. **(a)** The risk distribution between AS and healthy subjects, where AS has a much higher risk score than healthy samples. **(b)** ROC curve evaluated the discrimination ability of classifier model.

**Supplementary Figure** **2** Difference in the abundance of each immunocyte cell between healthy and AS samples.

**Supplementary Figure 3** Difference in the immune reaction activity between healthy and AS samples.

**Supplementary Figure 4** Difference in the expression of each HLA gene between healthy and AS samples.

**Supplementary Figure 5** 8 of 26 m6A regulators were differentially expressed in GSE25101 dataset.

**Supplementary Figure 6** 7 of 26 m6A regulators were differentially expressed in GSE181364 dataset.

**Supplementary Table S1** Detailed information of datasets.
